# Effects of Partial Substitution of Fish Meal by Soybean Meal with or without Heat-Killed *Lactobacillus plantarum* (LP20) on Growth Performance, Digestibility, and Immune Response of Amberjack, *Seriola dumerili* Juveniles

**DOI:** 10.1155/2015/514196

**Published:** 2015-02-01

**Authors:** Mahmoud A. O. Dawood, Shunsuke Koshio, Manabu Ishikawa, Saichiro Yokoyama

**Affiliations:** ^1^The United Graduate School of Agriculture Sciences, Kagoshima University, 1-21-24 Korimoto, Kagoshima 890-0056, Japan; ^2^Department of Aquaculture, Faculty of Aquatic and Fisheries Sciences, Kafrelsheikh University, Kafrelsheikh 33516, Egypt; ^3^Laboratory of Aquatic Animal Nutrition, Faculty of Fisheries, Kagoshima University, 4-50-20 Shimoarata, Kagoshima 890-0056, Japan

## Abstract

A 56-day feeding trial was conducted to evaluate the effects of supplemented diets with heat-killed *Lactobacillus plantarum* (HK-LP) with graded levels of soybean meal (SBM) on growth, digestibility, blood parameters, and immune response of *Seriola dumerili* (initial weight, 25.05 ± 0.1 g). Seven isonitrogenous and isolipidic practical diets were formulated to contain 0%, 15%, 30%, and 45% SBM, and each SBM level was supplemented with HK-LP at 0.0 and 0.1%. Fish fed diet which contains 30% SBM with HK-LP grew significantly faster than the other groups with notable feed intake and protein retention. Further, protein gain, whole body protein content, protease activity, protein, and lipid digestibility were significantly increased for all fish groups except for fish fed diet which contains 45% SBM with or without HK-LP. Interestingly, lysozyme activity was significantly enhanced in fish fed diets that contain 15% and 30% SBM with HK-LP. Hematocrit, peroxidase, and bactericidal activities revealed a significant increase in 30% SBM with HK-LP group. In addition, fish fed diets which contain 0% and 30% SBM with HK-LP showed higher tolerance against low-salinity stress compared with other groups. In conclusion, the addition of HK-LP to amberjack diets appeared to improve SBM utilization, immune response, and stress resistance.

## 1. Introduction

Fish meal (FM) represents an ideal nutritional source of dietary protein for fish. Increasing demand, unstable supply, and high prices of FM along with the continuous expansion of aquaculture are reasons for many nutritionists to realize that soon they will no longer be able to afford it as a major protein source in aquafeeds. Currently one of the challenges that fish nutritionists face is the need to partially or totally replace FM with less expensive, non-traditional animal or plant protein sources [1, 2].

Soybean proteins have been recognized as one of the most appropriate alternative protein sources for FM in aquafeed because of their consistent nutritional composition, comparatively balanced amino acid profile, availability, and reasonable price [3]. Soybean meal (SBM) has proven to be well accepted by yellowtail [4–6]. Tomás et al. [7] investigated the possible use of SBM as a substitute for FM in the diets of yellowtail by progressively increasing its inclusion level. The authors found a decrease in final weights as the SBM content increased starting from 30% protein substitution rate.

Methods for increasing SBM's inclusion rates in soy-sensitive species such as amberjack are required, and one of the methods is to apply dietary supplementation of functional compounds. Non-viable microbes exhibit beneficial effects due to their function as immunostimulants. Using live bacteria may cause a potential risk to wild aquatic organisms considering the fact the bacteria may escape into the environment. Therefore, the use of inactivated bacteria clearly solves such safety-related issues since they can no longer interact with other aquatic organisms [8]. Besides that, inactivated bacteria are considered one of the most practical candidates of feed additives. This is due to its high tolerance against temperatures which are produced during preparation of fish diets especially in the course of mincing and pelleting, without affecting its functional activity. This makes it more efficient than other candidates to achieve success in fish farming [8–10].

Heat-killed* Lactobacillus plantarum* (HK-LP) is a potential candidate as one of the functional additives for fish. Recently, effects of HK-LP have been investigated as immunostimulants [11–14]. Khonyoung and Yamauchi [14] reported that the diet supplemented with HK-LP (L-137) might activate intestinal function by increasing segmented filamentous bacteria, while inducing a better body weight gain in broilers. Oral administration of HK-LP has enhanced growth performance and immune responses of larval and postlarval Kuruma shrimp,* Marsupenaeus japonicus* bate [13, 15]. Oral administration of inactivated* Lactobacillus delbrueckii* subsp.* lactis *and* Bacillus subtilis* appears to cause good immune stimulatory properties of gilthead seabream (*Sparus aurata* L.) [8, 9]. Oral administration of heat-killed* Enterococcus faecalis* enhanced growth performance and immune responses of rainbow trout [10]. Furthermore, heat-killed bacteria were also compared with live form in tilapia [16]. According to these observations, it was hypothesized that HK-LP may also be effective in responses of growth and non-specific immune systems of amberjack,* Seriola dumerili*.

The amberjack is one of the most important cultured species in Japan because of its delicacy and comparatively higher market value. It is distributed throughout the tropical and subtropical seas except the Pacific Ocean [17, 18]. There have been no studies about dietary SBM and the effect of HK-LP have been undertaken on amberjack to date; the trial reported here was conducted to determine the effects of the partial substitution of FM by SBM with or without HK-LP on growth, digestibility, blood chemistry, immune responses, and stress resistance of amberjack juveniles.

## 2. Materials and Methods

### 2.1. Test Diets

Tables 1 and 2 show the composition and chemical analysis of the experimental diets. All the dietary components were obtained commercially, except for HK-LP preparation which was provided by House Wellness Foods Corp. (Itami, Japan) and it contains 20% HK-LP and 80% dextrin in dried-weight basis. HK-LP Prep (LP20) was prepared based on the method previously described by Murosaki et al. [19]. The product was stored at −20°C until use. Using brown fish meal and soybean meal as main protein sources and Pollack liver oil and soybean lecithin as main lipid source, seven isonitrogenous (50.5% crude protein) and isolipidic (12.3% crude lipid) practical diets were formulated to contain 0%, 15%, 30%, and 45% soybean meal and two levels of HK-LP (0.0 and 0.1%) (SBM0, SBM15, SBM15(0.1), SBM30, SBM30(0.1), SBM45, and SBM45(0.1)). Moreover, crystalline amino acid (CAA) mixture of lysine, methionine, betaine, glycine, and alanine were supplemented to meet essential amino acid (EAA) requirements of juvenile amberjack. Wheat flour was supplied as the carbohydrate or nitrogen-free extract source, activated gluten was used as a binder to produce pellet diet, and cellulose powder was used to adjust to 100% total proportion. The diets were prepared by thoroughly mixing all the dry ingredients in a food mixer for 15 minutes. Pollack liver oil, soybean lecithin, and HK-LP Prep were premixed with a sonicator (CA-4488Z, Kaijo Corporation, Tokyo, Japan), added to the dry ingredients, and mixed for another 15 min. Water (35–40% of the dry ingredients) was then added to the premixed ingredients and mixed for an additional 15 min. The pH of the diets was adjusted to the range of 7.0–7.5 with 4 N sodium hydroxide. The mixture was then passed through a meat grinder with an appropriate diameter (2.2–3.1 mm) to prepare pellets, which were then dried in a dry-air mechanical convection oven (DK 400, Yamato Scientific, Tokyo, Japan) at 50°C for about 120 min to approximately 10-11% moisture. The test diets were stored in a freezer at −20°C until use.

### 2.2. Experimental Fish and Feeding Protocol

Juvenile amberjack (*Seriola dumerili*), with mean initial body weight of 25.05 ± 0.1 g (mean ± S.E.), were purchased from Kagoshima prefecture seed production center, Kagoshima Prefecture, Japan, and transferred to the Kamoike Marine Production Laboratory, Faculty of Fisheries, Kagoshima University, Japan. The fish were acclimatized for two weeks in laboratory conditions and reared in a 500 L tank with flow-through system. During this period, a commercial diet (50% crude protein; Higashimaru, Japan) was supplied to the fish. Stocking was done at twenty fish per tank with the triplicate tanks per treatment in 200 L polycarbonate tanks (filled with 180 L of water) in a flow-through sea water system where each tank was equipped with an inlet, outlet, and continuous aeration. The tanks were maintained under natural light/dark regime. All fish were fed the respective test diets to satiation level by hand twice a day at 9.00 and 16.00 h., 7 days per week for 56 days. Any uneaten feed left was removed after feeding and dried using a freeze drier then subtracted from the total feed intake. The seawater was pumped from the deep basin of Kagoshima Bay, Japan. It was gravel-filtered and supplied into the system. A flow rate of 1.5 L min^−1^ was maintained throughout the experimental period. During the experimental period, the monitored water quality parameters (mean ± S.D.) were as follows, water temperature 25.2 ± 1.3°C, pH 8 ± 0.5, salinity 33.3 ± 0.5 ppt, and dissolved oxygen 6.1 ± 0.5 mg L^−1^. These ranges were considered within optimal values for juvenile amberjack.

### 2.3. Sample Collection and Biochemical Analysis

At the beginning, a pooled sample of 10 fish was stored at −20°C for initial whole body analysis. While at the end of the feeding trial, all fish were fasted for 24 h prior to final sampling. All the fish were anaesthetized with Eugenol (4-allylmethoxyphenol, Wako Pure Chemical Ind., Osaka, Japan) at 50 mg L^−1^. Then the total number, individual body weight, and length of fish from each tank were measured. Three fish from each replicate tank were randomly collected and stored at −20°C for final whole body analysis. Blood was taken from the caudal vein of five fish in each replicate tank using heparinized disposable syringes. A small fraction of the heparinized blood was used to analyze the hematocrit and hemoglobin levels. Hematocrit was determined using the microhematocrit technique. Plasma samples were obtained by centrifugation at 3000 ×g for 15 min at 4°C using a high-speed refrigerated microcentrifuge (MX-160; Tomy Tech USA Inc., Tokyo, Japan) and kept at −80°C. In addition, non-heparinized disposable syringes were used to collect blood for serum analysis. Serum samples were obtained by centrifugation at 3000 ×g for 15 min at 4°C to collect serum. Three other fish were randomly sampled from each dietary tank and used for collection of liver and viscera. Viscera and liver were removed then weighed to get viscerosomatic index (VSI) and hepatosomatic index (HSI), respectively. Digestive tracts were separated, cut into small pieces, washed with pure water, pooled together, and stored at −80°C.

Hemoglobin, plasma chemical parameters, and total serum protein (TSP) were measured spectrophotometrically with an automated analyzer (SPOTCHEM EZ model SP-4430, Arkray, Inc., Kyoto, Japan) [20]. Biological antioxidant potential (BAP) and reactive oxygen metabolites (d-ROMs) were also measured spectrophotometrically from blood plasma with an automated analyzer (FRAS4, Diacron International s.r.l., Grosseto, Italy) by following [21, 22]. Plasma cortisol was measured using commercial kits (Cortisol EIA Kit, product number EA65, Oxford Biomedical Research Inc., Oxford, MI) according to the procedure outlined by the manufacturer. Protease activity (PA) was analyzed using digestive organ samples according to Kader et al. [23].

The ingredients, diets, and fish whole body were analyzed for moisture, crude protein, total lipid, and ash, in triplicate, using standard methods [24]. This entailed moisture analysis by oven-drying at 110°C to constant weight, crude protein analysis by the Kjeldahl method, crude lipid analysis by the Soxhlet extraction method, and ash content analysis by combustion in Muffle furnace at 550°C for 4 h. The amino acid profiles of the experimental diets were analyzed by high performance liquid chromatography (HPLC, Shimadzu Corp. Kyoto, Japan) according to the previous studies [22, 25].

### 2.4. Low-Salinity Stress Test

Tolerance against exposure to low-salinity seawater was examined. After the feeding trial, five fish from each rearing tank (total of 15 fish per treatment) were randomly selected and transferred into a 100 L black tank containing low-salinity water (0.2%). The city tap water was dechlorinated by strongly aerating for at least 24 h and mixed with seawater, and then used as low-salinity water. The tanks for stress test were equipped with continuous aeration and kept under ambient temperature during the stress test. The number of dead fish in each test tank was recorded every 20 min. The passing of time to reach 50% death was calculated using the method previously described by [26, 27].

### 2.5. Evaluation of Non-Specific Immune Responses

Lysozyme activity of serum was determined with turbidimetric assays [28] at 450 nm with ImmunoMini NJ-2300 (System Instruments, Tokyo, Japan). A unit of enzyme activity was defined as the amount of enzyme that causes a decrease in absorbance of 0.001/min.

The serum bactericidal activity was measured according to Iida et al. [29]. Serum was diluted 3, 4, and 5 times with a Tris buffer (pH 7.5). The dilutions were mixed with a bacterial suspension (0.001 g/mL,* Escherichia coli*, IAM1239 cell line, Kagoshima, Japan) and incubated at 25°C for 24 h by microtube rotator (MTR-103, AS ONE, Osaka, Japan). The solutions were incubated on TSA (Trypto-Soya agar, Nissui Phatmaceutical Co. Ltd., Japan) at 25°C for 24 h. Colony forming unit (CFU) was counted by the plate counting method as described by Ren et al. [27].

The total peroxidase content in serum was measured according to Salinas et al. [9], with some modifications. Briefly, 15 *μ*L of serum was diluted with 35 *μ*L of Hank's buffered salt solution (HBSS) without Ca^+2^ or Mg^+2^ in flat-bottomed 96-well plates. Then, 50 *μ*L of peroxidase substrate (3, 30, 5, 50-tetramethylbenzidine hydrochloride) (TMB; Thermo Scientific Inc., USA) was added. The serum mixture was incubated for 15 min. The colour-developing reaction in serum samples was stopped by adding 50 *μ*L of 2 M sulphuric acid and the OD (450 nm) was measured in a plate reader. PBS was used as a blank instead of serum.

### 2.6. Digestibility Assessment

Digestibility of each diet was measured after the growth trial. For the digestibility measurement, remaining fish from the same treatments were distributed randomly into duplicate tanks. The fish were fed a diet containing chromium oxide (Wako Pure Chemical Industries, Ltd) as the inert marker at a level of 0.5% (Cr_2_O_3_, 5 g/kg) was added. Fish were acclimated to the diet containing chromic oxide for five days. In the morning of the 6th day, fish were fed each diet to apparent satiation twice daily. Six hours after feeding, feces were collected by putting pressure from belly to anus. Feces collection continued for ten days until a sufficient amount of feces had been collected for analysis. Pooled fecal samples were immediately ground after freeze-drying and kept at −20°C until analysis. Concentration of chromium oxide in diets and feces was determined according to Furukawa and Tsukahara [30].

### 2.7. Evaluation of Growth Performance Parameters

The following variables were evaluated:

 weight gain (%) = (final weight − initial weight) × 100/initial weight;
 specific growth rate (SGR %, day^−1^) = {(Ln(final weight) − Ln(initial weight))/duration (56 days)}  × 100;
 survival (%) = 100 × (final no. of fish/initial no. of fish);
 feed intake (FI, g fish^−1^ 56 days^−1^) = (dry diet given − dry remaining diet recovered)/no. of fish;
 feed efficiency ratio (FER) = live weight gain (g)/dry feed intake (g);
 protein efficiency ratio (PER) = live weight gain (g)/dry protein intake (g);
 protein gain (PG, g kg weight gain^−1^) = {(final weight (g) × final whole body protein content (%)/100) − (initial weight (g) × initial whole body protein content (%)/100)}/(weight gain (g)) × 1000;
 protein retention (PR, % of intake) = [protein gain (g kg weight gain^−1^) × 100]/protein intake (g kg weight gain^−1^);
 condition factor (CF) = weight of fish (g)/(length of fish)^3^ (cm)^3^ × 100;
 hepatosomatic index (HSI, %) = weight of liver/weight of fish × 100;
 viscerosomatic index (VSI, %) = weight of viscera/weight of fish × 100;
 apparent digestibility coefficient (ADC, %) = 100 − [(% Cr_2_O_3_ in diet/% Cr_2_O_3_ in feces) × (% nutrient in feces/% nutrient in diet)].

### 2.8. Statistical Analysis

All data were subjected to statistical verification using package super ANOVA 1.11, Abacus Concepts, Berkeley, California, USA. Probabilities of *P* < 0.05 were considered significant. Differences in significance between means were evaluated using the Turkey Kramer test.

## 3. Results

### 3.1. Growth Parameters and Nutrient Utilization

Growth performance and feed utilization of the fish are given in Table 3. Survival (%) of fish did not differ significantly (*P* > 0.05) among treatments. Final weight, weight gain (%), specific growth rate (SGR), and protein gain (PG) of fish fed SBM30(0.1) diet was significantly higher than those fed the other diets. On the other hand, the growth parameters of fish fed SBM15, SBM15(0.1), and SBM30 were not significantly different from those of fish fed FM (SBM0). The poorest growth performance was found in fish fed SBM45 and SBM45(0.1). However, SGR was significantly (*P* < 0.05) higher in fish fed SBM45(0.1) than SBM45. Similarly, PG and protein retention (PR) were also significantly decreased in fish fed SBM45 and SBM45(0.1) while no difference was detected between FM (SBM0) and the remaining treatments. However, no difference was detected in feed efficiency ratio (FER) and protein efficiency ratio (PER) between FM (SBM0) and other dietary groups.

Dietary treatments significantly affected (*P* < 0.05) feed intake (FI) of fish. FI was markedly improved by supplementing CAA and HK-LP Prep. Significantly higher FI was found in fish fed SBM30(0.1) compared to other diet groups. However, there were no significant differences in FI of fish fed SBM15, SBM15(0.1), SBM30, and FM (SBM0). On the other hand, SBM45 and SBM45(0.1) diets were not well accepted by the fish, and the value was significantly lower (*P* < 0.05) than other test diets.

### 3.2. Whole Body Proximate Analysis

The proximate composition of the whole body of juvenile amberjack is shown in Table 4. In comparison with the control, dietary treatments had no significant influence on the total lipid and crude ash contents at the end of the feeding trial. However, whole body crude protein contents in all experimental groups were significantly higher than SBM45 and SBM45(0.1) groups. Moreover, moisture content was significantly (*P* < 0.05) decreased in fish fed SBM0 and SBM15(0.1) groups. No difference (*P* > 0.05) was also detected in CF, HSI, and VSI of fish among treatments (Table 4).

### 3.3. Blood Parameters and Responses against Stress

Table 5 represents the blood parameters of amberjack after 56 days of feeding trial. Overall, dietary treatments had no effect on blood chemical parameters of fish except for the case of hematocrit, hemoglobin, glucose, and triglyceride (TG). Hematocrit level was significantly (*P* < 0.05) higher in fish fed SBM30(0.1) than those fed SBM30 diet while no significant (*P* > 0.05) differences were detected among other groups. Similarly, hemoglobin was significantly increased in fish fed SBM0 when compared with the SBM45 group while no significant differences were detected among other groups. Plasma glucose content was significantly (*P* < 0.05) more decreased in the SBM0 group than in other groups. On the other hand, TG was significantly (*P* < 0.05) higher in SBM0 than SBM30(0.1) group while no significant (*P* > 0.05) differences were detected among other groups. Experimental diets had no significant effect on the relative value (%) of plasma cortisol levels among all treatments.

Oxidative status of fish was analyzed from plasma (Table 5). The lowest values of reactive oxygen molecules (d-ROMs) were detected in fish fed SBM0, SBM15, and SBM30(0.1) diets. On the other hand, biological antioxidant potential (BAP) was found highest in the SBM30(0.1) group.  Figure 2 shows the pattern of combined effects of d-ROMs and BAP. The SBM0, SBM30, and SBM30(0.1) groups were located in zone (A), SBM15(0.1) group in zone (B), SBM15 and SBM45(0.1) in zone (C), and SBM45 group in zone (D), respectively.

**Figure 1:**
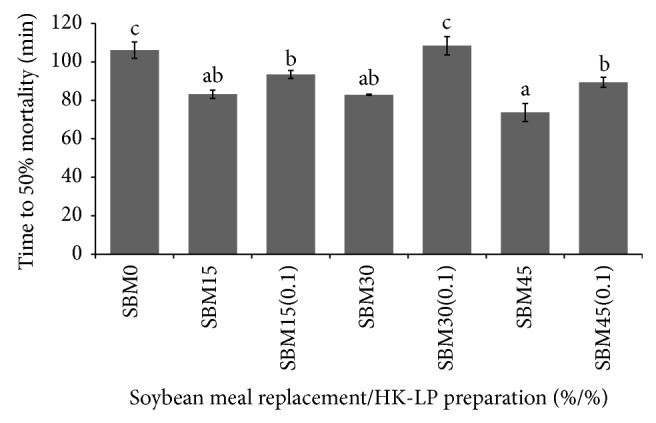
Time to 50% mortality (min) after low salinity stress test when amberjack fed increasing levels of SBM with or without the inclusion of HK-LP for 56 days. Values are means ± SE from triplicate groups. Means with different alphabet are significantly different (*P* < 0.05).

**Figure 2:**
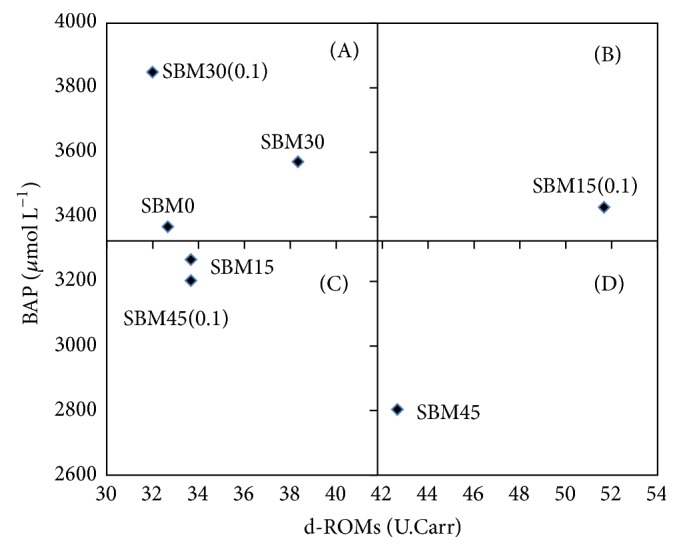
Oxidative stress parameters in amberjack fed test diets for 56 days. Values are expressed as mean ± SE (*n* = 3). Central axis based on mean values of d-ROMs and BAP from each treatment. Zone (A): high antioxidant potential and low reactive oxygen metabolites (good condition); Zone (B): high antioxidant potential and high reactive oxygen metabolites (acceptable condition); Zone (C): low antioxidant potential and low reactive oxygen metabolites (acceptable condition); Zone (D): low antioxidant potential and high reactive oxygen metabolites (stressed condition). Abbreviation used: SBM0, SBM15, SBM15(0.1), SBM30, SBM30(0.1), SBM 45, and SBM 45(0.1), respectively, refer to soybean replacement/HK-LP, %/%.

Figure 1 shows the results of the low-salinity stress test. The fish that received SBM0 and SBM30(0.1) diets clearly showed significantly (*P* < 0.05) higher tolerance against low-salinity stress than those of other groups. However, time to 50% mortality was found significantly the lowest in SBM45 group.

### 3.4. Non-Specific Immune Responses

**Figure 3:**
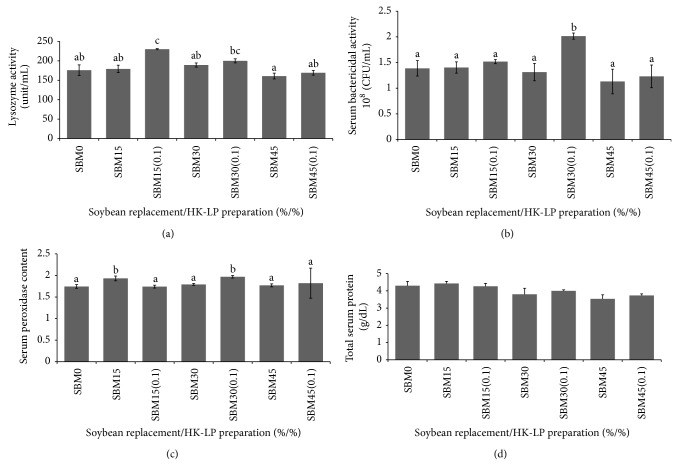
Immune parameters of amberjack juveniles fed diets containing increasing levels of SBM with or without the inclusion of HK-LP for 56 days. (a) Serum lysozyme activity (unit/mL, *n* = 3); (b) serum bactericidal activity (10^8^ cfu/mL, *n* = 3); (c) serum peroxidase activity (*n* = 9); (d) total serum protein (g/dL, *n* = 3). Data represent means ± SE. Values with the same letter are not significantly different (*P* > 0.05). Means with different alphabet are significantly different (*P* < 0.05). Absence of letters indicates no significant difference between treatments.

Figure 3 shows immune parameters after 56 days feeding trial. Serum lysozyme activity was significantly (*P* < 0.05) increased in the SBM15(0.1) group when compared with other groups while no significant difference was detected between the SBM15(0.1) and SBM30(0.1) groups (Figure 3(a)). Fish fed SBM30 diet supplemented with 1 g kg^−1^ HK-LP showed significantly higher serum bactericidal activity than the other groups (Figure 3(b)). Similarly, serum peroxidase activity recorded the highest significant values (*P* < 0.05) in the SBM15 and SBM30(0.1) groups (Figure 3(c)) while no significant differences were detected among other groups. Although not statistically significant, the comparatively higher total serum protein values were found in SBM0, SBM15, and SBM15(0.1) groups (Figure 3(d)).

### 3.5. Protease Activity and Digestibility Coefficients

Protease activity (PA, unit mg^−1^ protein) in the digestive tract of amberjack recorded no significant differences between SBM0, SBM15, SBM15(0.1), SBM30, and SBM30(0.1) groups. However, the SBM45 group recorded the poorest PA value among other experimental groups; moreover, the PA was significantly higher in the SBM45(0.1) group than the SBM45 group. The apparent digestibility coefficient (ADC) of protein was significantly (*P* < 0.05) higher in fish fed SBM0, SBM15, SBM15(0.1), SBM30, and SBM30(0.1) groups than SBM45 and SBM45(0.1) groups. ADC of lipid was found to be significantly different (*P* < 0.05) with being higher in the SBM15 and SBM30(0.1) groups than the other experimental groups (Table 6).

## 4. Discussion

Usually, lower feed intake could be the main reason for reduced growth performance when fish meal was replaced by soybean meal (SBM) [31]. However, the practical application of heat-killed* Lactobacillus plantarum* (HK-LP) to improve SBM utilization in amberjack diets represents a novel HK-LP application in the present study. Up to 30% SBM replacement level with amino acid mixture did not significantly reduce growth and feed utilization of amberjack. This was consistent with the findings of other previous studies in yellowtail [4–7]. Moreover, SBM30 diet supplemented with 1 g kg^−1^ HK-LP recorded the highest growth performance compared with other experimental diets. The beneficial effects of HK-LP supplementation on final body weight, weight gain, and specific growth rate of fish fed SBM30(0.1) diet were also found in the study of Tung et al. [13], who reported improved growth performance of kuruma shrimp* Marsupenaeus japonicus* fed with HK-LP. Growth promoting activity has been noted also in rainbow trout fed diet supplemented with heat-killed* Enterococcus faecalis* [10]. Significantly higher protein gain and protein retention in fish fed diet SBM30(0.1) would be a possible reason for the higher performances of fish in this group. These results suggest that the tested fish utilized experimental diets effectively by HK-LP supplementation resulting in increased feed intake in SBM30(0.1) group.

Several authors have reported that the dietary administration of different bacterial forms enhanced the secretion of intestinal enzymes and characterization of these enzymes provides some information regarding the digestive capacity of fish to hydrolyze carbohydrate, protein, and lipid of feed ingredients, leading to better growth performance and feed efficiency [32–35]. Khonyoung and Yamauchi [14] reported that the intestine is the direct organ for digestion, absorption, and immunity, as the gut microflora is continuously exposed to other strain of HK-LP (L-137). The latter was also thought to affect the production of extracellular enzymes by the microflora within the gastrointestinal (GI) tract of fish. The bacterial flora in the GI tract of fish shows very broad and variable enzymatic potential, and these enzymatic masses may positively affect the digestive process of fish [34, 36]. All together, the relatively enhanced growth performance and feed efficiency in the amberjack fingerlings fed the HK-LP supplemented diets could be related to the improved intestinal microbiota.

The protease activity (PA) of the digestive tract could provide further insight into the possible effects of different diets on fish performance [23]. In this study, PA was significantly enhanced in the SBM0, SBM15, SBM15(0.1), SBM30, and SBM30(0.1) groups compared to other experimental groups, while PA was significantly higher in fish fed SBM45(0.1) diet than that in fish fed SBM45 without HK-LP diet, indicating the positive effect of HK-LP. Similarly, it has been reported that the secretion of proteases was enhanced by supplementing immunostimulants in yellowtail,* Seriola quinqueradiata* diet [37]. Watanabe et al. [6] and Tomás et al. [7] reported that ADC of dry matter, protein, and lipid was high due to the process used for preparing diets in which pellets heating might have inactivated the trypsin inhibitor. Previous studies have also demonstrated that growth-promoting additives resulted in the improved digestibility of nutrients [10, 38]. Comparatively low digestibility values recorded here were likely due to the quality of raw material or due to the method of feces collection [39].

Blood parameters are important tools for indication of physiological stress response, general health conditions, and welfare of fish towards nutritional and environmental changes [40]. Blood parameters obtained in the present experiment are considered to be within the normal range for juvenile amberjack, compared to those of the previous findings [18, 41]. Results of the present study showed that the hematocrit values increased in the case of SBM30(0.1) group, implying improved health status. High hematocrit values indicate HK-LP efficiency, wherein iron is evenly distributed without any reduction in the synthesis of hemoglobin. Similarly, Rodriguez-Estrada et al. [10] reported that hematocrit level was enhanced by the supplementation of inactivated* Enterococcus faecalis* in rainbow trout diets. Moreover, lower triglyceride and cholesterol contents in fish fed SBM30(0.1) diet showed that the optimum availability of HK-LP in fish diets maintains low level of plasma triglycerides and cholesterol in fish.

Oxidative stress was measured using the free radical analytical system assessing the derivatives of oxidative stress by measuring reactive oxygen metabolites (d-ROMs test) and biological antioxidant potential (BAP test) in plasma samples. It is the consequence of an imbalance between oxidants and antioxidants in which oxidant activity exceeds the neutralizing capacity of antioxidants [42]. Recently, d-ROMs and BAP were reported to be reliable parameters for determining the oxidative stress conditions of fish [43]. It would be concluded that fish fed diets SBM0, SBM30, and SBM30(0.1) were in less oxidative stress conditions compared to the SBM45 group in this study.

The lethal stress test is used to assess the healthy status by measuring the lethal time of 50% mortality (LT_50_) in fresh water of the fish [25]. It is well known that stress affects the survival and growth of fish, since stress responses tend to increase the energy demand at the expense of anabolic processes [44]. The higher value of LT_50_ in the SBM0 and SBM30(0.1) groups indicated a higher tolerance of the amberjack against low-salinity stress. Fish antioxidant status is strongly related to immune system, contributing to enhance resistance towards different stressors [45]. In the light of the previous findings, results of the current study confirmed a higher tolerance against low-salinity stress in fish in less oxidative stress conditions.

Lysozyme is an important defense molecule of fish innate immune system [46]. Lysozyme activity has been used to evaluate the non-specific defense ability in many fish species, such as Japanese eel,* Anguilla japonica* [27], yellowtail kingfish,* Seriola lalandi* [47], and here Japanese flounder,* Paralichthys olivaceus* [48]. The lowest lysozyme activity was found in SBM45 group, which together with other parameters (bactericidal activity, peroxidase activity, and total serum protein) implied a less-healthy condition of the fish fed with this diet. The increasing trends in serum lysozyme activity in this study might have contributed to the enhancement in the non-specific defense mechanisms [49]. Serum bactericidal activity is one of the most important factors in host resistance against pathogenic bacteria [50]. In this study, the highest serum bactericidal activity was found in SBM30(0.1) group. Similarly, the highest levels of peroxidase were observed in the case of SBM15 and SBM30(0.1) confirming other results obtained by Salinas et al. [9]. From the mentioned results, it could be concluded that the non-specific immune response was enhanced by HK-LP supplementation. Similarly, Irianto and Austin [51] illustrated that dietary supplementation of inactivated bacteria also stimulated the innate immune parameters of rainbow trout,* Oncorhynchus mykiss*.

The potentials for reducing stress and enhancement of immunity and stress resistance by manipulation of nutritional factors and use of feed additives (such as HK-LP) were demonstrated in this study. However, very little work in this area has been conducted in fish. Thus, the effects of dietary functional feed additives and their interactions need to be assessed to develop economically viable feeds and feeding practices to optimize growth, improve stress resistance, immune response, and disease resistance and improve product quality of aquaculture species.

## 5. Conclusions

In conclusion, the present study shows that up to 30% SBM substitution level with essential amino acid supplementation did not significantly reduce growth, feed utilization, and immune response of amberjack. Furthermore, the addition of HK-LP to diets appeared to improve SBM utilization by amberjack. However, further studies are needed in order to evaluate the effects of HK-LP on amberjack health with attention to the intestinal microbiota and histology.

## Figures and Tables

**Table 1:** Formulation of the experimental diets (% dry matter).

Ingredient	Soybean meal (SBM) replacement, % (HK-LP Prep., %)						
	SBM0	SBM15	SBM15(0.1)	SBM30	SBM30(0.1)	SBM45	SBM45(0.1)
Brown fish meal^1^	61	51	51	40	40	30.5	30.5
Soybean meal^2^	0	15	15	30	30	45	45
Wheat flour	10	8	8	5	5	1	1
Soybean lecithin^3^	3	3	3	3	3	3	3
Pollack liver oil^4^	5	5	5	5	5	5	5
Vitamin mixture^5^	3	3	3	3	3	3	3
Mineral mixture^6^	3	3	3	3	3	3	3
Stay-C^7^	0.1	0.1	0.1	0.1	0.1	0.1	0.1
Activated gluten^8^	5	5	5	5	5	5	5
*α*-Cellulose	9	5.63	5.53	4.17	4.07	2.2	2.1
Amino acid premix^9^	0.9	1.27	1.27	1.73	1.73	2.2	2.2
HK-LP Prep^10^	0	0	0.1	0	0.1	0	0.1

Total	100	100	100	100	100	100	100

^1^Nihon Suisan Co. Ltd (Tokyo, Japan), ^2^J. Oil Mills, Japan, ^3,4^Riken Vitamins, Tokyo, Japan.

^5^Vitamin mixture (g kg^−1^ diet): *β*-carotene, 0.10; Vitamin D_3_, 0.01; Menadione NaHSO_3_·3H_2_O (K_3_), 0.05; DL-*α*-tochopheryl acetate (E), 0.38; thiamine-nitrate (B_1_), 0.06; riboflavin (B_2_), 0.19; pyridoxine-HCl (B_6_), 0.05; cyanocobalamin (B_12_), 0.0001; biotin, 0.01; inositol, 3.85; niacine (Nicotic acid), 0.77; Ca pantothenate, 0.27; folic acid, 0.01; choline chloride, 7.87; *ρ*-aminobenzoic acid, 0.38; cellulose, 1.92.

^6^Mineral mixture (g kg^−1^ diet): MgSO_4_, 5.07; Na_2_HPO_4_, 3.23; K_2_HPO_4_, 8.87; Fe citrate, 1.10; Ca lactate, 12.09; Al(OH)_3_, 0.01; ZnSO_4_, 0.13; CuSO_4_, 0.004; MnSO_4_, 0.03; Ca(IO_3_)_2_, 0.01; CoSO_4_, 0.04.

^7^L-ascrobil-2-phosphate-Mg.

^8^Glico Nutrition Company Ltd. Osaka, Japan. Commercial name: “A-glu SS”.

^9^Amino acid premix (g 100 g^−1^ diet) at soybean meal replacement level of 15%; the mixed amino acids just as follows: lysine, 0.20; methionine, 0.17; alanine, 0.30; betaine, 0.30; glycine, 0.30. Amino acid premix (g 100 g^−1^diet) at fish meal replacement level of 30%; the mixed amino acids just as follows: lysine, 0.46; methionine, 0.38; alanine, 0.30; betaine, 0.30; glycine, 0.30. Amino acid premix (g 100 g^−1^diet) at fish meal replacement level of 45%; the mixed amino acids just as follows: lysine, 0.72; methionine, 0.58; alanine, 0.30; betaine, 0.30; glycine, 0.30.

^10^HK-LP Prep: preparation of heat-killed *Lactobacillus plantarum* made by House Wellness Foods Corp. (Itami, Japan).

**Table 2:** Chemical analysis of the experimental diets.

Ingredient	Soybean meal (SBM) replacement, % (HK-LP Prep., %)						
	SBM0	SBM15	SBM15(0.1)	SBM30	SBM30(0.1)	SBM45	SBM45(0.1)
Proximate composition (%, dry matter basis)							
Crude protein	50.81	50.47	50.34	50.15	50.74	51.38	50.82
Total lipid	11.85	12.29	11.93	12.74	12.71	12.66	12.30
Ash	11.10	10.78	11.41	11.50	11.54	11.37	11.82
Gross energy (KJ g^−1^)^1^	19.75	19.92	19.63	19.84	19.91	19.95	19.76

Amino acid profiles (AA g 100 g^−1^ diet, dry matter basis)							
Arginine	2.81	2.94	2.86	3.02	2.93	3.17	3.01
Histidine	1.42	1.23	1.35	1.36	1.47	1.54	1.39
Isoleucine	2.26	2.24	2.04	2.38	2.18	2.27	2.47
Leucine	3.97	3.74	3.94	3.64	3.84	3.45	3.85
Lysine	3.91	3.81	3.61	3.75	3.66	3.89	3.59
Methionine	1.77	1.60	1.54	1.39	1.59	1.54	1.24
Phenylalanine	2.01	2.07	2.17	2.10	2.21	2.18	2.18
Threonine	2.18	2.26	2.26	2.11	2.11	2.01	2.29
Valine	2.55	2.76	2.66	2.52	2.62	2.46	2.36
ΣIDAA^2^	22.86	22.64	22.42	22.27	22.61	22.50	22.37

^1^Calculated using combustion values for protein, lipid, and carbohydrate of 23.6, 39.5, and 17.2 kJ g^−1^, respectively. Carbohydrate was calculated by the difference: 100 − (protein + lipid + ash + moisture).

^2^ΣIDAA: total indispensable amino acid contents.

**Table 3:** Growth parameters and nutrient utilization in amberjack fed test diets for 56 days^*^.

Parameters	Soybean meal (SBM) replacement, % (HK-LP Prep., %)						
	SBM0	SBM15	SBM15(0.1)	SBM30	SBM30(0.1)	SBM45	SBM45(0.1)
Fn wt^1^	136.6 ± 2.8^b^	136 ± 3.06^b^	136.7 ± 2.78^b^	136.9 ± 3.11^b^	149.2 ± 2.36^c^	101.7 ± 1.31^a^	105.8 ± 1.29^a^
WG^2^	445.1 ± 11.5^b^	443.8 ± 11.94^b^	446.4 ± 11.21^b^	446.8 ± 12.72^b^	494.1 ± 8.52^c^	304.8 ± 5.78^a^	320.95 ± 6.06^a^
SGR^3^	3.03 ± 0.04^c^	3.02 ± 0.04^c^	3.03 ± 0.04^c^	3.04 ± 0.04^c^	3.18 ± 0.03^d^	2.49 ± 0.03^a^	2.57 ± 0.02^b^
FI^4^	120.62 ± 3.06^b^	118.68 ± 3.19^b^	120.63 ± 1.56^b^	119.58 ± 2.39^b^	130.27 ± 2.6^c^	88.2 ± 4.24^a^	90.33 ± 2.68^a^
FER^5^	0.93 ± 0.03	0.94 ± 0.04	0.93 ± 0.02	0.94 ± 0.02	0.95 ± 0.03	0.87 ± 0.03	0.89 ± 0.03
PER^6^	1.82 ± 0.06	1.86 ± 0.08	1.83 ± 0.05	1.85 ± 0.04	1.87 ± 0.06	1.7 ± 0.06	1.76 ± 0.05
PG^7^	203.65 ± 1.88^b^	204.38 ± 5.08^b^	207.05 ± 3.69^b^	200.92 ± 1.22^b^	206.85 ± 2.01^b^	179.9 ± 1.64^a^	183.17 ± 0.2^a^
PR^8^	124.76 ± 2.57^b^	122.67 ± 6.1^b^	126.49 ± 2.19^b^	121.12 ± 3.55^b^	137.17 ± 1.93^c^	81.32 ± 3.26^a^	84.07 ± 2.29^a^
Sur^9^	100	95	100	100	100	90	93.33

^*^Values are means of triplicate groups ± S.E.M. Within a row, means with different letters are significantly different (*P* < 0.05); means with the same letters are not significantly different (*P* > 0.05). Absence of letters indicates no significant difference between treatments.

Average initial body weight; means ± S.E.M., 25.05 ± 0.1 g.

^1^Fn wt: final weight (g), ^2^WG: percent weight gain (%), ^3^SGR: specific growth rate (% day^−1^), ^4^FI: feed intake (g dry diet fish^−1^ 56 days^−1^), ^5^FER: feed efficiency ratio, ^6^PER: protein efficiency ratio, ^7^PG: protein gain (g kg body weight gain^−1^), ^8^PR: protein retention (% of intake), and ^9^Sur: survival (%).

**Table 4:** Whole body proximate analysis (%) and somatic parameters in juvenile amberjack fed test diets for 56 days^*^.

Parameters	Initial^1^	Soybean meal (SBM) replacement, % (HK-LP Prep., %)						
		SBM0	SBM15	SBM15(0.1)	SBM30	SBM30(0.1)	SBM45	SBM45(0.1)
Moisture	72.84	71.07 ± 0.2^a^	71.17 ± 0.31^ab^	71.02 ± 0.09^a^	71.18 ± 0.55^ab^	71.2 ± 0.2^ab^	72.44 ± 0.05^b^	72.44 ± 0.05^b^
Crude protein	19.58	20.22 ± 0.15^b^	20.27 ± 0.41^b^	20.5 ± 0.3^b^	20 ± 0.1^b^	20.5 ± 0.16^b^	18.38 ± 0.13^a^	18.62 ± 0.01^a^
Total lipid	3.42	4.48 ± 0.08	4.17 ± 0.14	4.35 ± 0.15	4.43 ± 0.15	4.26 ± 0.05	4.49 ± 0.01	4.33 ± 0.14
Crude ash	4.05	4.1 ± 0.09	3.95 ± 0.09	3.78 ± 0.02	3.96 ± 0.01	3.99 ± 0.05	3.75 ± 0.36	3.72 ± 0.17
CF^2^	—	1.41 ± 0.02	1.39 ± 0.04	1.37 ± 0.03	1.45 ± 0.03	1.4 ± 0.05	1.53 ± 0.04	1.39 ± 0.04
HSI^3^	—	1.1 ± 0.06	1.13 ± 0.12	1.08 ± 0.1	1.13 ± 0.06	1.04 ± 0.09	1.35 ± 0.01	1.13 ± 0.06
VSI^4^	—	3.75 ± 0.21	3.67 ± 0.12	3.65 ± 0.27	3.84 ± 0.07	4.04 ± 0.28	3.84 ± 0.05	3.78 ± 0.1

^*^Values are means of triplicate groups ± S.E.M. Within a row, means with different letters are significantly different (*P* < 0.05); means with the same letters are not significantly different (*P* > 0.05). Absence of letters indicates no significant difference between treatments. Crude protein, crude lipid, and ash are expressed on a wet weight basis.

^1^Initial values are not included in the statistical analysis.

^2^CF: condition factor (%), ^3^HSI: hepatosomatic index (%), and ^4^VSI: viscerosomatic index.

**Table 5:** Blood parameters in juvenile amberjack fed test diets for 56 days^*^.

Parameters	Soybean meal (SBM) replacement, % (HK-LP Prep., %)						
	SBM0	SBM15	SBM15(0.1)	SBM30	SBM30(0.1)	SBM45	SBM45(0.1)
Hematocrit (%)	48.7 ± 1.2^ab^	46.7 ± 0.7^ab^	47.3 ± 0.3^ab^	44 ± 1^a^	49.7 ± 1.7^b^	44.7 ± 0.9^ab^	46.7 ± 1.2^ab^
Hemoglobin (g/dL)	12.5 ± 0.00^b^	12 ± 0.3^ab^	12.3 ± 0.2^ab^	11.2 ± 0.8^ab^	12.2 ± 0.2^ab^	10.7 ± 0.2^a^	11.8 ± 0.1^ab^
Total protein (g/dL)	4 ± 0.1	3.6 ± 0.3	3.9 ± 0.03	4.1 ± 0.2	4.1 ± 0.3	3.6 ± 0.1	3.6 ± 0.1
Total bilirubin (mg/dL)	0.5 ± 0.1	0.4 ± 0.1	0.4 ± 0.1	0.7 ± 0.1	0.4 ± 0.1	0.4 ± 0.1	0.6 ± 0.2
Glucose (mg/dL)	68 ± 3.1^a^	93.3 ± 3.3^bc^	97.3 ± 1.2^c^	90.7 ± 3.2^bc^	89 ± 3.2^bc^	79 ± 4.6^ab^	85.7 ± 4.4^bc^
GOT (IU/l)^1^	35 ± 12.3	44 ± 8.7	39.7 ± 3.8	55.3 ± 7.8	40.3 ± 5.5	31.3 ± 3.7	53 ± 4.9
GPT (IU/l)^2^	<1.00	<1.00	<1.00	<1.00	<1.00	<1.00	<1.00
BUN (mg/dL)^3^	9 ± 0.6	8.7 ± 0.9	11.7 ± 0.9	9 ± 1.5	13.3 ± 1.5	11 ± 2	10.3 ± 0.3
TG (mg/dL)^4^	158 ± 9^b^	108 ± 5^ab^	145.3 ± 10.7^ab^	143.7 ± 22.9^ab^	93 ± 4.4^a^	123.3 ± 17.6^ab^	121 ± 6.5^ab^
T-Cho (mg/dL)^5^	238 ± 3.6	242.3 ± 25.8	290 ± 4	298.3 ± 29.7	255.3 ± 15.4	251.7 ± 20.5	229.3 ± 14.3
CORT (%)^6^	103.1 ± 3.1	101.3 ± 2.4	100.6 ± 1.4	102.2 ± 1.1	100 ± 2	110 ± 2.5	106.9 ± 4.9
d-ROMs^7^	32.7 ± 5.2^a^	33.7 ± 4.8^a^	51.7 ± 8.8^b^	38.3 ± 3.8^ab^	32 ± 2.3^a^	42.7 ± 3^ab^	33.7 ± 5.4^ab^
BAP^8^	3369.3 ± 84^ab^	3267.7 ± 169.9^ab^	3429.3 ± 335.6^ab^	3570.7 ± 16.9^ab^	3848 ± 92.5^b^	2803 ± 186.6^a^	3202.3 ± 108.3^ab^

^1^GOT: glutamyl oxaloacetic transaminase, ^2^GPT: glutamic-pyruvate transaminase, ^3^BUN: blood urea nitrogen, ^4^TG: triglyceride, ^5^T-Cho: total cholesterol, ^6^CORT (%): relative value of cortisol, ^7^d-ROMs: reactive oxygen metabolites, and ^8^BAP: biological antioxidant potential.

^*^Values are means of triplicate groups ± S.E.M. Within a row, means with different letters are significantly different (*P* < 0.05); means with the same letters are not significantly different (*P* > 0.05). Absence of letters indicates no significant difference between treatments.

**Table 6:** Protease activity (PA, unit mg^−1^ protein) in the digestive tract and apparent digestibility coefficients (ADC) in amberjack fed test diets^*^.

Parameters	Soybean meal (SBM) replacement, % (HK-LP Prep., %)						
	SBM0	SBM15	SBM15(0.1)	SBM30	SBM30(0.1)	SBM45	SBM45(0.1)
PA (unit mg^−1^ protein)^1^	0.039 ± 0.001^c^	0.04 ± 0.002^c^	0.041 ± 0.001^c^	0.04 ± 0.001^c^	0.043 ± 0.003^c^	0.029 ± 0.001^a^	0.035 ± 0.001^b^
ADC_Protein_^2^	90.01 ± 0.17^b^	90.77 ± 0.48^b^	89.68 ± 0.6^b^	90.11 ± 0.42^b^	91.34 ± 0.94^b^	86.93 ± 0.69^a^	87.59 ± 0.69^a^
ADC_Lipid_^3^	85.39 ± 0.63^b^	89.51 ± 0.23^c^	85.78 ± 0.63^b^	86.76 ± 0.85^b^	88.78 ± 0.5^c^	82.32 ± 0.49^a^	83.26 ± 0.55^a^

^*^Values are means of triplicate groups ± S.E.M. Within a row, means with different letters are significantly different (*P* < 0.05); means with the same letters are not significantly different (*P* > 0.05).

^1^Protease activity (PA, unit mg^−1^ protein) in the digestive tract; apparent digestibility coefficients (ADC %). ^2^For crude protein; ^3^for lipid, respectively.
